# Applying and Assessing Participatory Approaches in an Environmental Flows Case Study

**DOI:** 10.1007/s00267-023-01829-6

**Published:** 2023-05-25

**Authors:** Meghan Mussehl, J. Angus Webb, Avril Horne, Libby Rumpff, LeRoy Poff

**Affiliations:** 1 Environmental Hydrology and Water Resources Group, Department of Infrastructure Engineering, The University of Melbourne, Melbourne, VIC Australia; 2 School of Ecosystem and Forest Sciences, The University of Melbourne, Melbourne, VIC Australia; 3 Department of Biology, Colorado State University, Fort Collins, CO USA; 4 Institute for Applied Ecology, University of Canberra, Canberra, ACT Australia

**Keywords:** Stakeholder engagement, Participatory methods, Structured decision-making, Environmental flows, Environmental water, Goulburn River

## Abstract

Environmental flows (e-flows) management takes place within a complex social-ecological system, necessitating the involvement of diverse stakeholders and an appreciation of a range of perspectives and knowledge types. It is widely accepted that incorporating participatory methods into environmental flows decision-making will allow stakeholders to become meaningfully involved, improving potential solutions, and fostering social legitimacy. However, due to substantial structural barriers, implementing participatory approaches can be difficult for water managers. This paper assesses the effectiveness of an e-flows methodology that combines elements of structured decision-making and participatory modeling, whilst constrained by project resources. Three process-based objectives were identified by the group at the start of the process: improving transparency, knowledge exchange, and community ownership. We evaluated the success of the approach according to those objectives using semi-structured interviews and thematic analysis. In evaluating how well the participatory approach achieved the process objectives, we found that at least 80% of respondents expressed positive sentiment in every category (*n* = 15). We demonstrate that the values-based process objectives defined by the participant group are an effective tool for evaluating participatory success. This paper highlights that participatory approaches can be effective even in resource-constrained environments when the process is adapted to fit the decision-making context.

## Introduction

Water is valued by people in many ways, ranging from the biophysical to the metaphysical (Anderson et al. 2019). This means a diverse range of stakeholders are concerned with the equitable and efficient stewardship of water resources. Abstraction, climate change, and other anthropogenic pressures have caused deterioration of freshwater social-ecological systems globally (Dudgeon et al. 2006, Vörösmarty et al. 2010). Environmental flows (hereafter e-flows) have emerged as a tool to restore and maintain freshwater systems. The Brisbane Declaration on Environmental Flows explicitly connects e-flows to human cultures, economies, and livelihoods, recognizing that healthy freshwater ecosystems are critical to developing sustainable, resilient communities and preserving cultural and natural heritage (Arthington et al. 2018). The Declaration goes on to outline the importance of local knowledge and diverse representation in decision-making for e-flows and identifies participation as an actionable recommendation within e-flows programs. However, a lack of effective community engagement and social acceptance has been a major barrier to the implementation of e-flows programs (Le Quesne et al. 2010, Horne et al. 2017, Harwood et al. 2018). Given the highly contested nature of riverine social-ecological systems and the range of relevant knowledge systems and values, it has been suggested that e-flows programs need to be built around robust and flexible participatory approaches (Cook et al. 2013a, Conallin et al. 2018) that recognize and incorporate a multitude of actors and perspectives (Conallin et al. 2017, Webb et al. 2018). In the last several decades there has been a shift toward implementing participatory decision-making approaches in water management, and within natural resource management contexts more broadly (Cook et al. 2013b).

There are three primary motivations for agencies and governments to implement participatory programs: normative, substantive, and instrumental (Krueger et al. 2016, Ricart et al. 2019). The *normative* motivation is driven by the belief that stakeholders should be able to influence policies and processes that affect them. This is a key component of deliberative democracy and underscores the importance of representation within decision-making (Stoll-Kleemann and Welp 2006, Stringer et al. 2006). The *substantive* motivation asserts that including a wider breadth of perspectives and knowledge results in better solutions and management strategies, ideally produced through a process of knowledge co-production (Wyborn 2015, van der Molen 2018). This idea is a critical to how adaptive management has been conceptualized within the context of e-flows (Allan and Watts 2018, Webb et al. 2018), which promotes engagement and participation to increase opportunities for both social and scientific learning (Stringer et al. 2006). The *instrumental* motivation aims to ensure public acceptance and perceived social legitimacy of water governance agencies (Irvin and Stansbury 2004, van Buuren et al. 2019). In the complex and sometimes volatile context of e-flows, social legitimacy has been identified as crucial to the ongoing success of these programs and a critical foundation for adaptive management (Gearey and Jeffrey 2006, Horne et al. 2017, O’Donnell et al. 2019).

Top-down, highly technocratic approaches to e-flows management often fail to gain widespread community support and thus may lack social legitimacy. Technocratic approaches are ingrained in regulatory frameworks and codified into policy, but these approaches leave little room for the integration of community knowledge systems when emphasis is placed on scientific “objectivity” and the process depends upon expert guidance (Godden and Ison 2019, Colloff and Pittock 2019). For example, even the Ecological Limits of Hydrologic Alteration (ELOHA) framework, which explicitly acknowledges the social dimensions of e-flows, still centers on flow-ecology relationships and prioritizes this type of knowledge (Poff et al. 2010, Pahl-Wostl et al. 2013, Anderson et al. 2019).

Participatory approaches that allow participants to shape the values and objectives of e-flows programs and engage in knowledge co-production create *input legitimacy* (Hogl et al. 2012, van Buuren et al. 2012). While e-flows programs frequently focus on the development of *output legitimacy* by demonstrating the ecological effectiveness of e-flows implementation, input legitimacy is built through transparency, access, and representation within decision-making (O’Donnell et al. 2019). These goals may be achieved by implementing intentional engagement strategies for e-flows decision making.

While it is widely accepted that stakeholder engagement is necessary within e-flows programs (with some instances of this being legislated (e.g., The Australian Water Act 2007), there are many barriers to the successful implementation of participatory methods. Water agencies operate within legal and regulatory frameworks with specific objectives and norms (Godden and Ison 2019, van Buuren et al. 2019). Within this operating framework, features such as project timelines and budget constraints can limit an agency’s ability to meaningfully engage with stakeholders (Irvin and Stansbury 2004). Participatory methods carry with them an inherent risk, as there is no guarantee that the process will go smoothly or end in consensus. Engaging with community members may alter project timelines and in worst-case scenarios, result in deadlock or create new conflicts (Reed 2008, Klijn and Koppenjan 2000). When agencies have legal obligations within institutional structures, it can be difficult to delineate the scope of participation and allow participants to have meaningful input into decision making (van Buuren et al. 2019). Agencies and water managers may avoid or limit participatory approaches because they lack the skills to navigate conflict, particularly when they themselves are an active stakeholder in the management process (Fisher et al. 2020). Designing an effective engagement strategy requires skilled facilitation, flexibility, and genuine intent.

Despite an emphasis on participatory approaches within the water management literature, there has not been widespread adoption of these methods or achievement of the expected benefits (Conallin et al. 2017). Participatory approaches are perceived as complex and resource intensive for organizers and participants (Roque et al. 2022), and it is unclear if projects with limited resources can benefit from investing in participatory approaches. The ultimate goal of evaluating the participatory process is to learn from past mistakes and adjust course to improve future participation (Rowe and Frewer 2000). The literature provides examples of meta-analyses that use post-hoc evaluate the effectiveness of participatory case studies (Falconi and Palmer 2017, Kovács et al. 2017). These analyses developed evaluation criterion that look at both the participatory *process* and the related *outcomes*, typically through the lens of credibility, salience, and legitimacy (Cash et al. 2002, Carr et al. 2012). Comparative evaluations have allowed researchers to identify characteristic of successful participation that have been used to develop best practice guidelines (Webler and Tuler 2002). However, these approaches do not give e-flows managers the tools to assess their own participatory programs in real time. Evaluating the success of these programs allows stakeholders to engage in social learning and make necessary changes to engagement strategies (Carr 2015). Further, evaluating the success of these programs provides justification for their ongoing implementation. Therefore, we developed a context-specific method for assessing participatory success within an e-flows assessment.

An e-flows assessment is the process of determining socio-ecological objectives for a river, bringing together the necessary knowledge and tools to understand the system, and ultimately providing recommendations about the flow regimes necessary to achieve these objectives (Arthington et al. 2018). E-flows assessments should be well-documented to be able to guide water managers in ongoing decision-making. E-flows assessments are fundamental to e-flows decision-making and are a key aspect of adaptive management of e-flows (Webb et al. 2018). In the last 20 years, a suite of tools and methodologies have been developed to guide the assessment process (Horne et al. 2017). Many of these methods focus on enhancing the understanding of ecological relationships in the system and prioritize technical and biophysical knowledge of the system. Holistic methodologies for e-flows assessments have identified the need for robust stakeholder engagement, but often fall short of guiding managers in the development of adequate participatory methods (Poff and Matthews 2013). Structured inclusion of participatory approaches with adequate evaluation will support learning within adaptive management cycles (Allan and Watts 2018, Conallin et al. 2018).

In this paper, we outline a process for evaluating the success of a participatory approach implemented during an e-flows assessment in Victoria, Australia. The e-flows assessment blended elements of structured decision-making and participatory modeling (Horne et al. 2022, Mussehl et al. 2022). Our purpose in this paper is not to present the participatory methodology itself, but to focus on the evaluation and assessment of participation. Process objectives focus on how decisions should be made and clarifies group values around decision-making (Keeney 1996, Gregory et al. 2012). Participant interviews and qualitative analysis were used to answer the question of how well the participatory approach achieved the process objectives defined by the participatory group. Our evaluation method was tailored to participant values and allowed us to demonstrate the benefits of the participatory approach.

## Methods

**Fig. 1:**
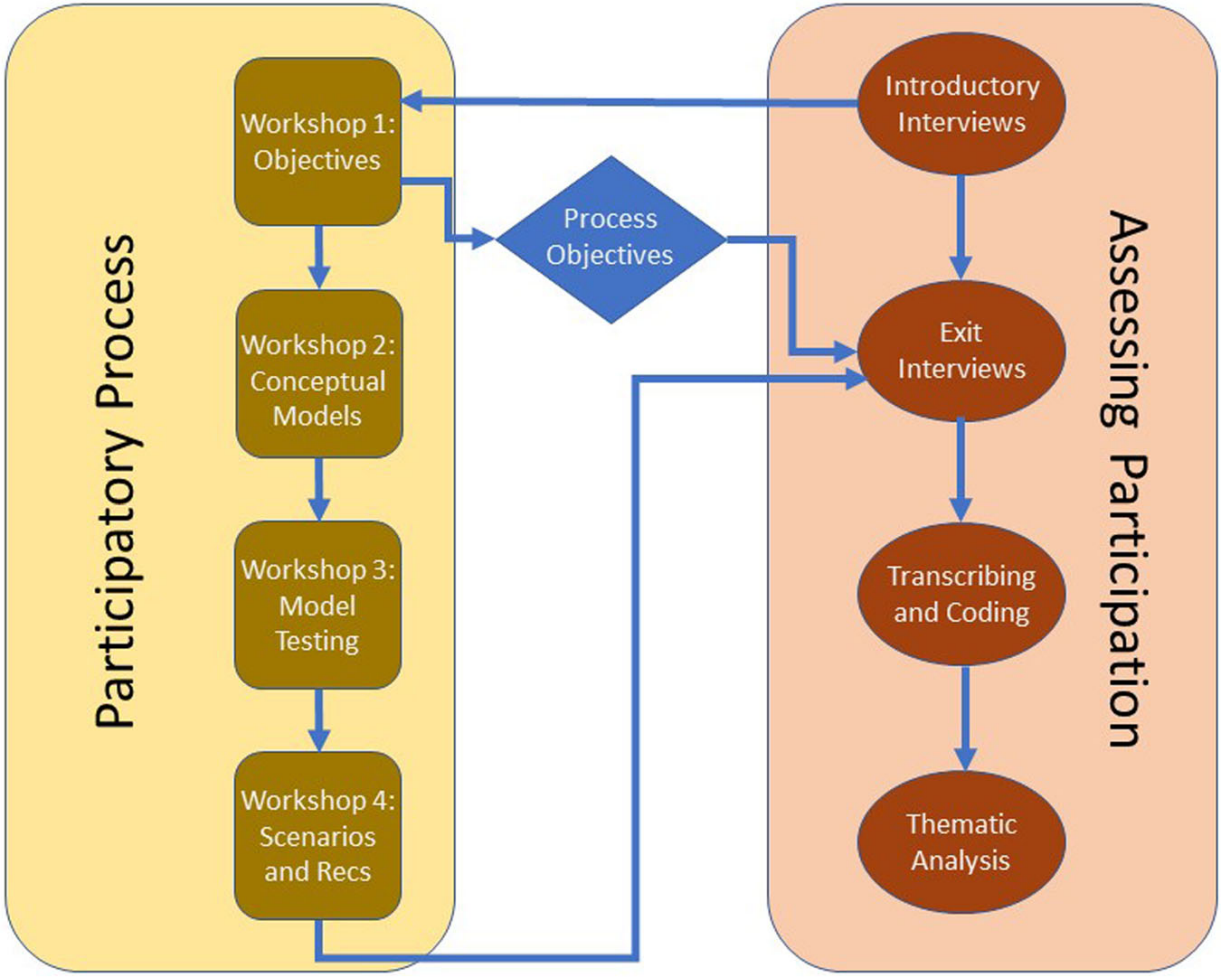
Schematic showing how the participatory process (yellow) described in Horne et al. 2022 and the assessment process (red) described in this paper are connected. Further details about the participatory process and workshops can be found in online Supplementary Material

We used a case study on the Kaiela (Lower Goulburn) River in Victoria, Australia to evaluate the success of a participatory process. The participatory process integrated participatory modeling into a structured decision-making framework to contribute to the development of e-flows recommendations (Gregory et al. 2012, Hemming et al. 2022). The assessment of participation took place in parallel to the project’s participatory process. While the participatory and assessment processes were distinct from one another (Fig. 1), these processes are interconnected. The purpose of this section is to detail the assessment process (shown in red in Fig. 1) used to evaluate the participatory process (shown in yellow). Further detail regarding the participatory process and case study can be found in Horne et al. (2020, 2022) and in online supplementary materials.

The participatory approach was structured around a series of workshops attended by diverse stakeholder groups from within the catchment area. These workshops were held in person from 2019–2020. The assessment process was built around semi-structured interviews with stakeholder participants. We conducted introductory interviews before the first workshop and exit interviews following the delivery of the final flow recommendations.

Structured decision-making is a formalized framework for decision-making that ensures the process is deliberate, defensible, and transparent (Hemming et al. 2022). Structured decision-making helps us clarify complexity in the process and define its critical features. One crucial benefit of this framework is articulating the connection between stakeholder values and the decision-making process (Keeney 1996).

Evaluation of the participatory success was based on participant responses regarding process objectives identified during the first workshop. Process objectives are used within structured decision-making to articulate the values of the participant group about the decision-making process itself (Gregory et al. 2012). Using the internally developed process objectives for our assessment meant the participants were able to define for themselves what successful participation looked like.

### Decision Context

**Fig. 2:**
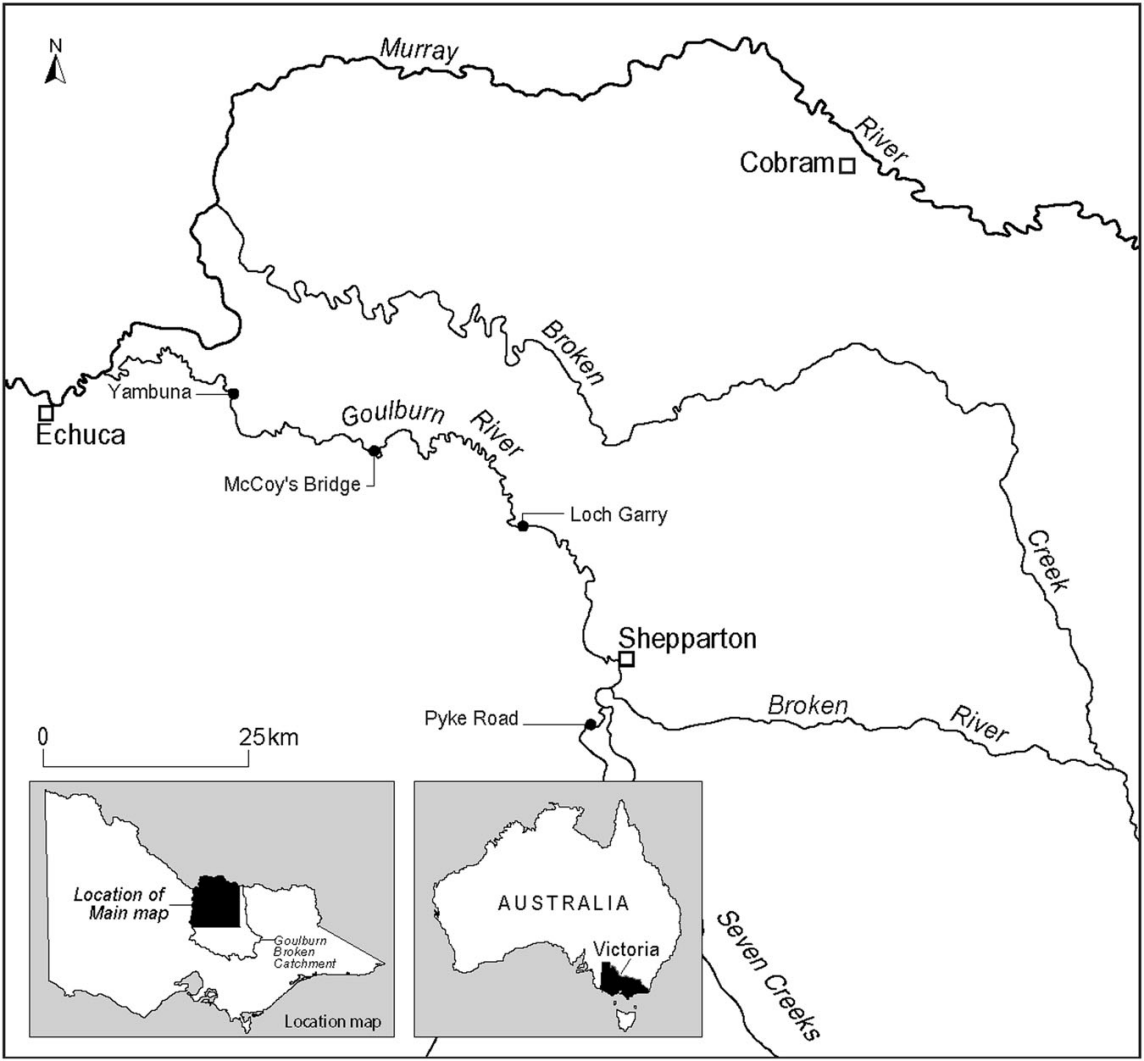
Kaiela (Lower Goulburn) River and surrounding areas (Reproduced from Webb et al. 2022)

The Kaiela (Lower Goulburn) River is a large, lowland river located within the Northern Victorian region of the Murray Darling Basin (MDB) in Australia (Fig. 2). The Kaiela is within the traditional lands of the Yorta Yorta Nation and comprises the section of river from Goulburn Weir to the confluence with the Murray River (see Fig. 2). Upstream of this section, from Goulburn Weir to Lake Eildon, the river is known as the Warring or Mid Goulburn River (part of the Taungurung Nation). Flows through the Kaiela are highly regulated by operations at Lake Eildon and Goulburn Weir. With much of the water flowing through the Warring diverted at Goulburn Weir for irrigation, winter, and spring flows in the Kaiela are lower than they would be under natural conditions. Transfers of irrigation and environmental water out of the Goulburn catchment also result in higher than natural summer flows (Treadwell et al. 2021).

Due to the presence of several socially and culturally significant species (e.g., platypus, turtles, River Red Gums), the Kaiela River has been identified as a high priority waterway (Victorian Waterway Management Strategy 2013). Environmental flows targeted at protecting critical habitat and restoring river condition have been delivered in the catchment since before 2012 and are managed through a regulatory framework that includes the local catchment management authority, state and federal agencies that hold entitlements to environmental water, and the regional water storage manager (Goulburn Broken Regional Catchment Strategy 2013, Treadwell et al. 2021). Entitlements for environmental water are water rights that can be actively managed and ordered from storage to support environmental outcomes (Doolan et al. 2017). In 2020, the e-flows assessment was updated with new recommendations (Horne et al. 2020) and that project served as the case study for the participatory process evaluated in this paper.

### Stakeholders

The stakeholder participants were recruited with guidance from the Goulburn-Broken Catchment Management Authority (GBCMA) and included four distinct stakeholder groups: local Indigenous organizations, community members, water agency representatives, and discipline-based researchers. Community member participants were recruited primarily from the GBCMA’s Environmental Water Advisory Group (EWAG). For the purposes of data analysis within this paper, Indigenous representatives from Yorta Yorta and Taungurung Aboriginal corporations were categorized as community members to protect their anonymity given the small participant group. Researcher stakeholders are members of the expert panel recruited to support the model development and provide a discipline-specific knowledge base for the development of flow recommendations. They participated in the workshop series as stakeholders and provided guidance for the development and quantification of supporting models. Water agency representatives from local, state, and federal levels included environmental management and infrastructure operators.

### Introductory Interviews

Before the first workshop, we conducted semi-structured interviews with members of the stakeholder participant group. Semi-structured interviews are comprised of both specific and open-ended questions, which allows researchers to investigate subjective perspectives and experiences utilizing a consistent framework (Bradford and Cullen 2012, Magaldi and Berler 2020). Interviews took place over the phone and were digitally recorded. Draft transcripts were generated using an online AI tool, Otter.ai (Otter 2022). Transcripts generated with this tool were then reviewed by the lead author and edited to reflect the accuracy of the digital recording. In total, 20 introductory interviews were conducted consisting of 8 community members, 7 water agency representatives, and 5 expert panel members.

A guiding set of questions was developed and used for all interviews (see online supplementary materials). Questions from the introductory interview targeted the participants’ pre-existing relationship with and knowledge of the catchment, including their experience with participatory engagement and decision making. The introductory interviews were used to create a baseline understanding of the participants’ knowledge and values.

### Workshop Series

The workshop series was the primary mode of engaging with stakeholder participants for the e-flows assessment. While ideally these workshops would have occurred in person, the COVID pandemic and strict lockdown requirements in Australia mandated that two of the four workshops be held online. The meetings included a mix of formal presentations, open Q&A, and breakout groups focused on specific activities. A breakdown of the workshop series including participants can be seen in Table 1.

**Table 1:** Breakdown of the participatory workshop series including participants and aims

	Format	Participants	Workshop Aims
Workshop 1	In-Person	Community Members Indigenous Reps Agency Reps Expert Panel Members	• Develop a shared understanding of the decision context • Understand relevant values • Define objectives
Workshop 2	In-Person	Community Members Indigenous Reps Agency Reps Expert Panel Members	• Review and finalize objectives • Develop conceptual models for ecological objectives
Workshop 3	Online	Expert Panel Members Agency Reps	• Validate quantitative models • Discuss flow recommendation development
Workshop 4	Online	Community Members Agency Reps	• Reengagement following COVID/Review of project status • Discuss/refine flow recommendations • Identify conflicts within flow recommendations • Discuss final report preparation

### Process Objectives

Three process objectives were elicited from and defined by stakeholders in Workshop 1 based on the objectives framework defined within the structured decision-making literature (Keeney 1996, Gregory et al. 2012). Process objectives were distinct from the fundamental and means objectives identified for the river during the workshop (Table 2). The project objectives were developed through facilitated activities during the workshop and refined through participant discussions. All objectives for this project were defined and clarified in the final e-flows assessment report, an summary of which can be found in online Supplementary Materials. The process objectives served as the foundation for the assessment methodology and thematic analysis. Importantly, these process objectives were identified by the participants themselves, and therefore provide a strong basis from which to assess success for the project.

**Table 2:** Process objectives with summary definition

Process objective	Summary definition
Community Ownership	Opportunities for meaningful community engagement and ensuring the representation is equitable. Community participants expect to be engaged during the entire project.
Knowledge Exchange	Decision process should include multidirectional learning. Community members have their own unique knowledge and perspectives of the river that need to be incorporated in the process.
Transparency	Detailed documentation of methodologies and decision-making that is accessible and communicated appropriately to all stakeholders.

### Exit Interviews

Exit interviews were conducted at the end of the project, after the final report and recommendations were submitted. These interviews took place over the phone or online and were digitally recorded. The same process was used to generate transcripts for these interviews. In total, 15 exit interviews were obtained: 5 community members, 5 water agency representatives, and 5 expert panel members. Not all participants from the introductory interviews participated in exit interviews. Exit interviews were more difficult to obtain once the project had ended.

Exit interview questions were also semi-structured and participants were encouraged to elaborate on their answers. However, questions were designed to target the process objectives developed in the first workshop. The exit interview questions were clearly divided into four sections, each with 4–6 questions aimed at each process objective and a final section about their overall experience.

### Analysis

Both introductory and exit interviews were analyzed using thematic analysis in NVIVO 12 Pro (NVivo 12 Pro 2020). Thematic analysis is common in qualitative research and allows for the systematic exploration of patterns and themes in textual data (Forman and Damschroder 2007, Clarke and Braun 2017). Initially, we used an inductive approach to analyze the introductory interviews. Introductory interview transcripts were read closely several times to identify the main themes and develop a thematic/coding framework. Codes are simplified labels that can be assigned to a segment of text within the data (Chandra and Shang 2019). After generating codes through multiple readings, a codebook was generated and used to explore patterns and identify overarching themes.

The process objectives were used to develop a coding framework that was then used to reassess the introductory interviews and analyze the exit interviews. This deductive coding method is a top-down approach, where an external framework or set of hypotheses is used to examine and categorize the data (Elo and Kyngäs 2008). This was particularly useful for analyzing the exit interview data, as the interview questions were structured around the process objectives. Using the same deductive framework for both the introductory and exit interview data allows us to characterize participants’ perception of the process objectives before the workshops and assess the project’s success in relation to these themes. To ascertain the perceived success of the process in relation to each of the process objectives, we used sentiment analysis within the coding framework. For example, the following interview question was developed to assess the transparency of the project.

Interview Question: Were you adequately updated about progress during the course of this project?

Participant Answer: Yeah, look, I think so, I don’t think I missed any meeting. If I had missed meetings, I think it would have been more difficult to keep up with things.

The participant’s answer was coded within the “transparency theme” and as having “positive sentiment.” We then assessed the proportion of positive or negative sentiments according to each theme.

## Results

### Introductory Interviews

The thematic analysis revealed that the process-based objectives featured heavily in the introductory interviews (Table 3). Community ownership and related issues around community support involvement were brought frequently across all stakeholder groups.

**Table 3:** Frequency of process-based objectives raised in introductory interviews

Theme	% of time spent on theme throughout introductory interviews^a^	# CM who mentioned theme	# Researchers who mention theme	# WA reps who mention theme
Community ownership	21	7	5	7
Knowledge exchange	12	7	4	4
Transparency	5	3	2	5

*CM* Community members, *WA* Water agency representatives

^a^Note interviews covered some aspects that moved away from these themes and hence this does not add to 100

### Community Ownership

All respondents discussed the idea of community ownership or engagement in the introductory interviews, frequently emphasizing its importance to the overall project.All participant groups expressed the importance of including a broad representation of stakeholders when including community in the decision-making process. Community members and water agency representatives highlighted the importance of community engagement during objective setting and expressed the desire to create a “shared understanding” or “vision” for the catchment. The view was expressed that community values should inform the selection of ecological objectives and that community members needed to have the opportunity to communicate their desires for the river. This desire for inclusivity, representation and access reflects the importance of establishing input legitimacy at the very beginning of a project (Table 4).

**Table 4:** Examples of participant quotes for community representation and objective setting

Key quotes on community representation and objective setting		
*Community members*	*Water agency representatives*	*Researchers*
“we need to be careful that we are inclusive of all, all views. I understand some views are going to carry more weight. But it’s important that all views are at least heard”	“identification of other values that might not necessarily come out through normal technical studies, that is a priority to the community consideration.”	“identifying what values and assets are important…whatever they value in the system is what we should be striving for”
“Anyone who is active user of the river and has that sort of local insight”		“they’ve [community members] definitely got a role right at the visioning stage…we need to, with the community, work out–what do we want, not assuming we know what they want.”
“the community often need to make the scientists aware of what desires they have for the river”		

Water agency representatives described the importance of developing community trust and buy-in for environmental watering programs. They recognized that to build up community support, community members needed to be meaningfully engaged with the process. Water agency representatives and researchers were more likely to explicitly refer to community buy-in, while community members referred to community support. There was a perception that better community engagement would help improve community opinion regarding e-flows. Respondents across all groups raised the concept of community champions, describing how knowledgeable community members involved in the process might become advocates within the broader community (Table 5).

**Table 5:** Examples of participant quotes on the importance of community support

Key quotes on community support		
*Community members*	*Water agency representatives*	*Researchers*
“if they don’t do it [community engagement], the whole process would be a total failure…because it wouldn’t have the confidence of the community”	“[if] there was no community consultation or input whatsoever- It just wouldn’t work. We’re not going to. Yeah, you’re just not going to have the support of the community and the social license to do what we do in terms of environmental flows”	“The approach gives the best the best chance of having community input through key representatives and having having those people being able to at least advocate for the process that we’ve gone through whether they agree with the outcomes or not.”
“in case you don’t have the support of the community…you won’t be able to implement anything”	“hopefully, then they’ll be able to communicate their support for it more broadly, in the community.”	“I think it could help in terms of community buy-in to the whole process.”

### Knowledge Exchange

Knowledge exchange was mentioned in 70% of introductory interviews (Table 2), with all community member participants discussing knowledge exchange or learning in some form.

Several community members expressed that they were looking forward to the opportunity to learn from other stakeholders. Community members were interested in learning about the ecology of the river, as well as the decision-making around e-flows. They saw the workshops as an opportunity to better understand how various stakeholders were engaged in this process. A few discipline researchers and agency representatives expressed that community members needed to be educated to better understand and engage with the process. Agency representatives expressed that engagement is a learning process and the participatory activities present an opportunity for community members to identify areas about which they are concerned, or questions that they have about the system, and to initiate novel conversations (Table 6).

**Table 6:** Examples of participant quotes for learning opportunities

Key quotes on learning opportunities		
*Community members*	*Water agency representatives*	*Researchers*
“I particularly like your expert panel. Yeah. I like learning from them.”	“I think it’s gonna be a good learning process that we can maybe refine over time and potentially apply to other similar projects.”	“And then it’s an opportunity for the members of the community to ask questions and whatnot. So that’s, that’s probably the extent of my involvement in that kind of stuff.”
“I’m keen to learn some of the other stakeholders and how they operate and how they function within, within the network”	“educating those members who might not have, I don’t know who they are, have a strong understanding of how, what is managed and the implications and restrictions on things that they might seem simple and easy to, to do in terms of flow management.”	“I think the community needs to first be educated and have a general understanding of the principles of flow and the ecology of different aspects of this system.”

The importance of community values and perspectives was acknowledged by all stakeholder groups and has been coded within this work under the theme of community ownership. However, some water agency representatives and most community members also went on to describe the benefits of community knowledge of the river. This community knowledge was described as place-based and derived from living in close proximity to the river over long periods of time. Some agency representatives expressed that community members might reveal unexpected insight. Most researchers and other agency representatives focused exclusively on community values and did not mention community knowledge of the river system (Table 7).

**Table 7:** Examples of participant quotes for the benefits of community knowledge

Key quotes on community knowledge		
*Community members*	*Water agency representatives*	*Researchers*
“I know a lot, a lot of stuff that you don’t see on paper, I can walk to the river to and tell you bits and pieces or I can reflect on different times”	“they’re providing local knowledge, insight into the local community values and ideas”	“I think identifying the community links to ecological values.”
“they’re trying to engage those sort of people who are on the river every day, and they know what’s going on. Because the CMA obviously can’t be everywhere.”	“I’m sure we get some insights and knowledge that we might not necessarily aware of, or pick up through normal processes….it can certainly broaden our scope and understanding of how the system operates and the issues”	“we need to know what the values are that the community has. And that requires participation and also learning so we will have to have our shared learning of our knowledge of the system.”

### Transparency and Trust

Transparency and trust were not as strong a theme as community ownership and knowledge exchange, with only two experts and three community members mentioning the issue explicitly. However, five out of the six water agency representative interviews acknowledged transparency and trust as essential to the process.

While agency representatives emphasized transparency explicitly, community members were much more likely to discuss “genuine intent”. There was a sense amongst community members that participatory processes can be “tokenistic” or are just “lip-service” practices designed to satisfy a requirement but nothing else. When community members felt agency representatives had genuine intent, they believed they would have influence in the process. Most community members expressed a positive outlook about the GBCMA’s intent and the upcoming workshops. Agency representatives acknowledged that trust and transparency was critical to the overall success of engagement and described the engagement as a long-term process geared toward building trust (Table 8).

**Table 8:** Examples of participant quotes for transparency, trust, and genuine intent

Key quotes on transparency, trust, and genuine intent		
*Community members*	*Water agency representatives*	*Researchers*
“I think they’re genuinely interested in a positive outcome….I think it’s a real attempt to gain or incorporate local knowledge”.	“It’s when you bring the community along for a journey, and make them feel that they’re involved in actually have really input to the process.”	“really being transparent. You know, if you do try, and I don’t know, skew things in one type of favor then the public are very quick to pick up on it. And then there’s a lot of mistrust. So as a scientist, trust is a very important thing.”
“shiny colored pamphlet that they put in front of you, with maps and all the rest of the stuff. You feel like that. They’re just telling you what’s going to be done and you might be able to influence it in a minor way.”	You do have to be prepared to spend a fair bit of time. So if you look at the first day or two of doing it, you think, well, what did we get done- not much. It is a slow process. It’s about building trust, that some of that trust is already done. We’re not starting from scratch.	

Water agency representatives and researchers both identified the importance of clarifying participants’ roles and potential for impact. This included explaining the process very clearly with the opportunities for community input identified. The importance of balancing expectations arose multiple times, with the acknowledgement that these expectations and interests may differ among stakeholders. Water agency representatives occasionally brought up previous experiences with community engagement to emphasize this importance, and there is a sense that past community dissatisfaction with e-flows may be due to a lack of clarity of their role within decision-making processes (Table 9).

**Table 9:** Examples of participant quotes for clarifying roles and expectations

Key quotes on transparency and clarity		
*Community members*	*Water agency representatives*	*Researchers*
No community members discussed the importance of clarify roles and expectations	“process needs to be clearly spelled out for participants, so they know how and when they can participate in how their information will feed in and be incorporated into the planning process.”	“setting expectations, everyone upfront on what that what that’s what processes and how it will be run… there should be no surprises”
	“so I think it’s sort of really about balancing community expectations and stakeholder expectations, I suppose. Everyone, sort of has a certain interest I suppose in the river”	

### Exit Interviews

#### Community Ownership

While all group participants were invited to participate in exit interviews, there were 15 that agreed to be interviewed (five individuals from each stakeholder group). Questions related to community ownership targeted the following themes identified during the introductory interview and workshops:

- Appropriate representation of stakeholders
- Process reflected community and group values
- Level of involvement for community members
- Improved relationships

**Fig. 3:**
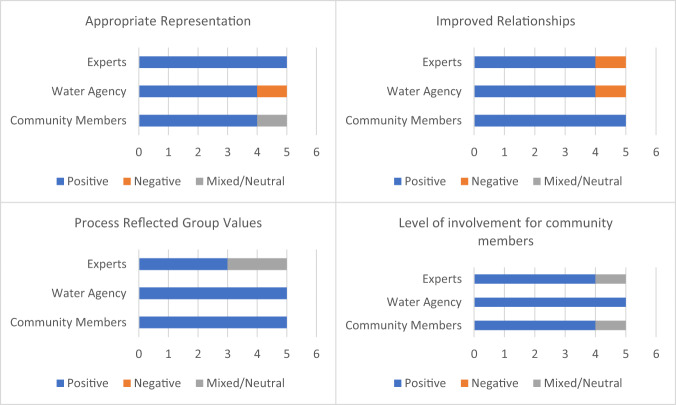
Sentiment breakdown of exit interview responses for key aspects of community ownership. For all stakeholder categories *n* = 5. For full question wording, see online Supplementary Materials

Responses across these four themes indicate that the participatory approach used in the project supported community ownership and input legitimacy (See Fig. 3). participants agreed there was an appropriate representation of stakeholders, though a lack of cultural and age diversity was noted. All stakeholder groups noted that it can be difficult to sustain engagement throughout the life of a project and that including too many stakeholders can complicate the process. They felt that the number of participants was appropriate for the success of the project.

There was a strong positive response from 13 of the 15 participants when asked whether they felt the workshops and project had helped foster relationships amongst stakeholders. Respondents across all stakeholder groups emphasized the importance of being present together and participating in conversations and noted that COVID restrictions impacted later stages of the project. Community members expressed that they felt their views had been included in the final report, though one noted that some technical aspects were difficult to engage with. Researchers were more likely to express neutral or mixed sentiments regarding community ownership, remarking that they felt they were unsure about this aspect of the project (Table 10).

**Table 10:** Examples of participant quotations from exit interviews regarding key themes within community ownership

	Key quotes on community ownership		
	*Community members*	*Water agency representatives*	*Researchers*
Appropriate representation	“Yeah, I thought you did pretty well. It’s always hard to get attendance. I would have liked to see more of the local Indigenous community.”	“Yeah, I think I think we had a good cross section of people there. I guess. I don’t know if it’s good, bad or indifferent, but it is the people that we’ve typically engaged with previously.”	“There’s certainly a wide variety of people there. But if there were people in groups that weren’t representative weren’t representative, I’m not sure-it seemed to be okay, but can’t give a definitive answer.”
Process reflected group values	“And we were able to bring up a lot of issues people feel strongly about, the overbank flows, the IVTs and big issues that need to be tackled in the future. To me that takes us forward.”	“So I think that the values the way that the fundamental objectives were written, actually reflected the community values a little bit better than I’d seen in a lot of the flow studies.”	“Yes, a lot of effort was clearly devoted to ensuring that this occurred.”
Improved relationships	“the process improved my understanding on where people were coming from”	“brought everyone together and that agency staff are able to actually hear it directly from community members to create a shared understanding”.	“having those conversations and getting to understand people’s thoughts and their values about flows, the importance of the flows for them”.
Level of involvement for community members	“I felt like I took a bit of a backseat during the modeling, but I felt like in the beginning, the community’s perspective was there.”	“I think early on yes. I found it hard towards the end. Obviously COVID impacted things. It was hard to the community reaction or input being put into the final product towards the end, especially once the meetings were online. Hard to see their input in the technical work”	Researchers did not elaborate on this theme

### Knowledge Exchange

**Fig. 4:**
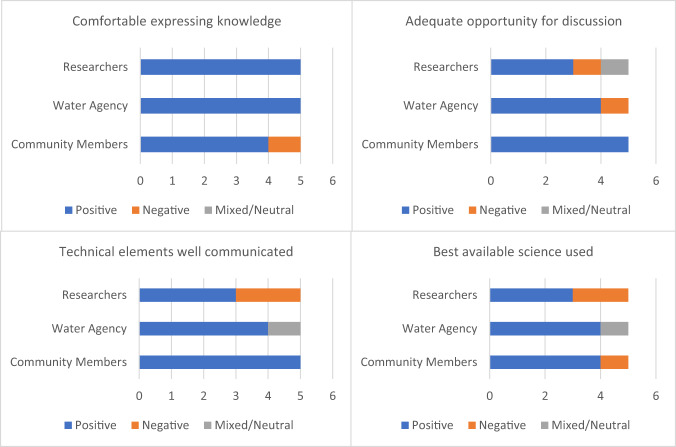
Sentiment breakdown of exit interview responses for key aspects of knowledge exchange. For all stakeholder categories *n* = 5. For full question wording, see online Supplementary Materials

Questions related to knowledge exchange targeted the following themes identified during the introductory interview and workshops (Fig. 4):

- Comfort expressing perspective and knowledge
- Adequate opportunity for discussion/questions
- Communication of technical project elements
- Use of “best available science”

All community members felt there was adequate opportunity to discuss the topics and issues with other stakeholders, while agency representatives and researchers had mixed responses. Three respondents noted the difficulty of discussing the project through the online platforms necessitated by the COVID pandemic. When asked whether the scientific elements were adequately communicated, all five community members responded positively. Most community members expressed that while they may not have completely understood all the technical material, they felt they understood the process on a conceptual level. This sentiment was further reflected by three water agency representatives, who noted that model development could be complex and difficult to understand. There was a sense that detailed understanding of technical elements is less important than understanding the process at a high level (Table 11).

**Table 11:** Examples of participant quotations from exit interviews regarding key themes within knowledge exchange

	Key quotes on knowledge exchange		
	*Community members*	*Water agency representatives*	*Researchers*
Comfortable expressing knowledge	“Yeah, that was really good. Providing a platform for sharing information was good”	“I did. No problems with that here. I think one thing that worked well was the way we split up into groups. And I think the community was able to have input into those groups. I think that was a good way to communicate.”	“Yep, for sure, I’m not a shrinking violet.”
Adequate opportunity for discussion	“a great opportunity” “didn’t leave any meeting with a bunch of questions I still needed to ask”	“there was a lot for participants to absorb, particularly for community members” “If I hadn’t been so familiar with the program, I might have been a bit more frustrated by not getting to dig into the discussion”.	“I think sometimes it was a little rushed…but the process was still really good. People did get the chance to have input”
Communication of technical elements	“to me, there’s no surprises or anything I didn’t understand”.	“the outcomes that were coming out [of the models] were meaningful, which I think boosts the community confidence in it”.	“I think the non-expert stakeholders appreciated the opportunity to interact with experts in developing the conceptual models.”
Use of “best available science”	“I have confidence that the science that was around the table was good- we made a very good selection” while another emphasized “we had the right kind of people in the room”. “we had the right kind of people in the room”.	“the latest science is a very fluid concept…but this process teased out that science a lot better with the ecological response models in a transparent way”.	“best available science on the Goulburn, yes. The best available science in developing flow recommendations from experience in other risks in other river systems, probably not.”

### Transparency and Trust

**Fig. 5:**
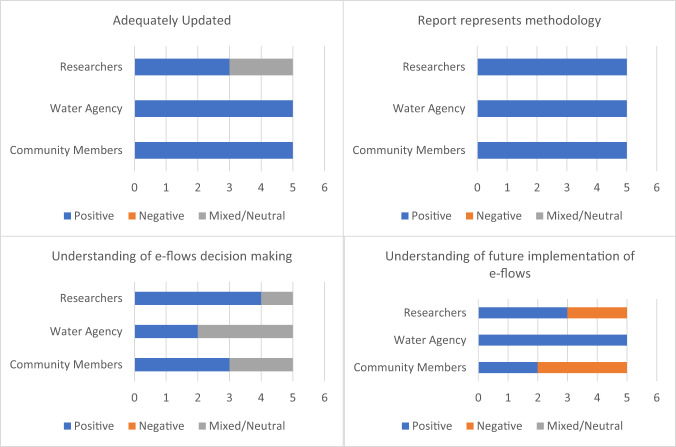
Sentiment breakdown of exit interview responses for key aspects of transparency and trust. For all stakeholder categories *n* = 5. For full question wording, see online Supplementary Materials

Questions related to transparency and trust targeted the following themes identified during the introductory interview and workshops (Fig. 5):

- Appropriate briefing and adequate updates during project
- Report represents methodology
- Understanding of e-flows decision making
- Understanding of future implementation of e-flows

Negative responses regarding transparency arose primarily from two questions: *“Do you have a good understanding of how these environmental flow recommendations will be applied in the future?”* and *“Has your understanding of environmental flows decision making improved?”* Both expert panel (researcher) members and community members believed that future application and implementation was dependent on “politics”. They describe a lack of transparency around internal decision making at these higher organizational levels. Community members, who started with a relatively lower understanding of these institutional dynamics, reported that their understanding had improved through their engagement with the project. However, another community member said that while the CMA and local level was very clear and transparent, there is a lack of clarity around decision making beyond the local level (Table 12).

**Table 12:** Examples of participant quotations from exit interviews regarding key themes within transparency and trust

	Key quotes on transparency and trust		
	*Community members*	*Water agency representatives*	*Researchers*
Appropriately briefed and updated	“I suspect the first meeting, I had to clarify whether we were locked into government policy or not. So I think the answer to this first question is- I could have been better briefed up front. As far as– that sort of area of the project.”	Agency representatives responded positively and did not elaborate	“Excellent in early stages but a bit patchy toward final stages, probably reflecting impact of COVID”
Report represents methodology	“I thought we achieved what we set out to achieve and with the wrap up, I was very pleased with the whole process.” “I mean our objectives are probably different from yours, those community objectives. And I think they were all expressed clearly in the meetings and I think the report reflects that.”	“There was some challenges for me between the modeling work that was done and the participatory approach to the flows study and how those two fit together. That part probably could have been a better done, how those things tie in together.”	Researchers responded positively and did not elaborate
Understanding of e-flows decision making	“I’ve come to a better understanding of just who can influence change and who didn’t. Who was locked into procedures and who wasn’t.” “I’ve got questions about the basin level. How are they making decisions around environmental flows?”	Agency representatives responded positively/neutrally due to pre-existing understanding of institutional decision-making.	“I suspect it’s more political realities and desires of certain politicians and downstream users. And that might totally override what has been recommended. And that’s just a real politic view.”
Understanding of future implementation of e-flows	“I don’t think anyone could honestly say… actual implementation, is really reliant on politics”	Agency representatives responded positively and did not elaborate	“I would be keen to get an update on how the CMA now uses that new understanding to make decisions on environmental watering, such as some examples of running the model to assess various flow events to deliver.”

### Overall Success

When asked about their overall experience, 13 participants responded that they had a positive experience through their engagement with the project. All community members and water agency representatives expressed positive opinions about the process. General feedback about the project was positive with water agency representatives and community members expressing satisfaction with the process and final product. Researchers were divided, with three expressing that they had positive experiences and enjoyed interacting with other stakeholders and developing the models. The negative responses from researchers were motivated by the expert-opinion elicitation process, which was not considered part of the project’s participatory process.

We asked participants to reflect on how the project was impacted by COVID and whether the project team responded adequately. All stakeholder groups expressed disappointment that the number of in-person workshops had to be limited. Several participants expressed that online workshops were not conducive to free-flowing conversation and that digital spaces were more tiring, necessitating shorter workshops. The online format limits a participant’s ability to engage with others and makes it more difficult to focus on complex material. One researcher described face-to-face interactions as *“more efficient, effective, and enjoyable… [than online]. You don’t chat with people over coffee, or over lunch. And often, that’s where the most interesting interactions occur”* (Table 13).

**Table 13:** Examples of participant quotes for overall feedback

Key quotes on overall success		
*Community members*	*Water agency representatives*	*Researchers*
“Look, I think it was a good process, you bent over backwards to be clear at every point. In the end, I think the community thought it was forward and well-articulated and argued. I think from the community point of view it’s really helpful to see that in the end there’s some real substance behind the concerns that they have.”	“I guess my comment is that I was really impressed. You know, it was different take on the flows study and it was really interesting to see the process that we went through, and there were definitely parts of the method that were important and other bits went a differently- I really liked the big workshop and the way the community was engaged. I think you might get differing opinions from the ecologists who are used to a certain way, but it’s interesting because when you look at the results, you end up getting a similar product. It brings everyone along the ride, it’s more inclusive than other flow studies I’ve been involved on. Credit to you guys, I think this one went well”	“Only that I’m very much in favor of multi group participation in environmental decision making, it shouldn’t be a technocracy or even worse. And it is driven by political concerns rather than best understanding.”

## Discussion

There are three primary motivations for adopting participatory approaches: the normative, substantive, and instrumental (Stirling 2008, Ricart et al. 2019). Within our project, we have observed how these three motivations influence the engagement process. Water managers and researchers discussed transparency, clarity, roles, and responsibilities, reflecting a concern with process and procedure. They emphasized the instrumental goal of community buy-in, using it as shorthand for social legitimacy. By contrast, community members described the desire to be heard, to engage with the process meaningfully, and be able to influence decision-making. This indicated that community members were more concerned with substantive and normative motivations.

While community ownership was the idealized goal of the engagement process, it is an ambiguous and difficult-to-define objective (Lachapelle 2008). Our definition of community ownership included “ownership of decisions” and emphasized participant involvement and representation in the entire decision-making process (Horne et al. 2020). Representation is a critical part of building legitimacy, contributing to community ownership through creating a sense of equity and fairness within the decision-making process (O’Donnell et al. 2019). All stakeholder groups in our project recognized the right of communities and individuals to have an influence on decisions that impact them, which reflects a normative motivation for including participation in e-flows projects (Ricart et al. 2019).

In our interviews, we saw major differences in how trust and transparency were articulated, reflecting divides between the stakeholder groups and their motivations. Researchers almost never mentioned the issue of trust unless explicitly asked. Trust is difficult to define, yet widely recognized as critical to building social legitimacy for e-flows (Stern and Coleman 2015, O’Donnell et al. 2019). We identified transparency as a process-based objective in our first workshop and elaborated in the final report on the importance of information sharing and clear communication of the process (Horne et al. 2020). Trust and transparency should be viewed through the lens of input legitimacy, as the level of trust is indicative of the ways we communicate and the relationships between stakeholders (O’Donnell et al. 2019). Without trust, participatory processes have no social legitimacy, input or otherwise. Under these conditions, e-flows programs will be stalled and decision-making will have no community support.

While transparency and accountability build towards *procedural trust*, this is not the only kind of trust that needs to be considered within engagement (Stern and Coleman 2015). This was reflected in our interviews, with water agency representatives likely to discuss transparency, but community members more likely to emphasize genuine intent. This reflects the necessity of *affinitive trust* or the trust a participant has for an individual representative’s motivations and concerns. Affinitive trust is built through iterative interactions with individuals perceived as having integrity (Stern and Coleman 2015). Our project benefitted from pre-existing relationships between the stakeholder participants. Key staff at the GBCMA have invested time and resources into building relationships with both community and researcher stakeholders over an extended period. This pre-existing level of trust was apparent in both introductory and exit interviews, but it is unclear if this trust is based at the individual level or if it extends to the GBCMA as an organization.

Communication and engagement are impacted by the scale of decision-making, and this is reflected in the trust community participants have for different institutional organizations (O’Donnell et al. 2019). While there is a great deal of trust for the local agency, our participants communicated that there was a “black box” beyond the local level and there was a perceived lack of transparency at the state and basin levels. Engaging with these agencies requires a high level of institutional understanding, even when transparency has been considered within their decision-making. Highly complex regulatory frameworks and organizations, such as those found in Australia, represent an additional barrier to meaningful community participation (Godden and Ison 2019).

Engaging stakeholder participants throughout the e-flows assessment resulted in significant differences in both the process and the outcomes compared to other traditional assessment approaches. Most importantly, how the project objectives were developed. Besides the inclusion of process-based objectives, which are not typically part of a traditional e-flows assessment, new fundamental ecological objectives were also selected. Driven by participant interest, the project included out-of-scope flows that would connect the floodplain to the river. Water representatives highlighted during interviews the inclusion of out-of-scope flows as beneficial to their overall planning and have used the results of the assessment to support negotiations aimed at relaxing flooding constraints. Participants were able to meaningfully influence decision-making within the project because water agency representatives were open to an exploratory process. This emphasizes the importance of including non-agency stakeholders in conversations around project scope and limitations. Allowing stakeholders to interrogate these boundaries can potentially reveal novel management strategies. Open, flexible processes that allow stakeholders to influence the decision-making process and substantially impact outcomes foster community acceptance of programs and build social legitimacy.

## Conclusion

Within this participatory process, we found that conceptualization of knowledge and participant roles in knowledge exchange varied between stakeholder groups. While community members and agency representatives valued community knowledge about the river, researchers were unlikely to express this sentiment. Furthermore, in introductory interviews, some researchers expressed hesitation about including community members in some aspects of the project, citing concerns that community members might not have adequate background or that the ecological objectives would not be a high priority. Given that researchers often have considerable experience with technical, top-down approaches to e-flows assessments, it will take time to shift the culture around how different types of knowledge are valued and expand the role of community participation (Rosendahl et al. 2015, Anderson et al. 2019). This shift can be facilitated by water managers and interdisciplinary researchers who design and implement participatory approaches, not only in e-flows but in all areas of natural resource management. Project development should include opportunities for agency representatives, community members, and scientific experts to have meaningful and challenging conversations. These transformative interactions between stakeholders are critical to shifting perspectives and fostering the conditions for new modes of knowledge creation.

While our analysis suggests that the participatory process was generally successful, several components of the project could have been improved. Due to a tight timeline, participant recruitment was largely drawn from the CMA’s pre-existing advisory group. While this ensured that most participants had a baseline knowledge of e-flows, there is also the possibility that the group was biased, and some perspectives were not represented. We would recommend dedicating more time to recruitment and casting a wide net to find potential participants. This may require additional resources as project team members would need to raise awareness around the project and have conversations within the community. Within this project, one team member was responsible for designing the participatory process and conducting all participant interviews. Upon project completion, agency representatives noted that this work was instrumental for achieving a high level of participation. Many projects would not have the staff capacity to dedicate to this process. Designing and implementing participatory approaches require specific skills and resources but can be effectively included through adequate resourcing and planning.

Working as a group to define the process-based objectives created the space to explore and articulate shared values and establish a baseline for communication amongst stakeholders. It also gave us a valuable tool for measuring participatory effectiveness with an internally defined metric. These objectives were clearly defined, communicated within the group, and presented in intermediate and final reports. Self-reflective participatory objectives are not a standard procedure in traditional e-flows assessments. By contrast, using process-based objectives to evaluate the success of an e-flows project enables self-reflective learning and can improve engagement strategies over time. It is essential that these objectives are defined by the participant group themselves and reflect the project context and group values. We recommend working collaboratively to clearly articulate these objectives, including definitions and methods of measurement.

Our project demonstrates that participation can be effective, even when subject to the budget and time constraints that typically limit the potential for meaningful stakeholder engagement. In our evaluation, respondents across stakeholder groups expressed positive sentiments regarding project outcomes for all three process-based objectives. Agencies and e-flows practitioners need to be aware of the differing motivations for a participant’s involvement in a project. If community stakeholders want to have a substantive impact on a project, then agencies need to facilitate these kinds of opportunities and approach participation with flexibility and openness. Collaborative development of process-based objectives opens these kinds of discussions and provides a self-reflective evaluation tool that can improve engagement over time. E-flows programs that include intentional, reflective participation approaches will allow stakeholders to build trust, explore new management strategies, and ultimately foster social legitimacy.

## Supplementary information

Supplementary materials
