# Supplementary material for: Applying and Assessing Participatory Approaches in an Environmental Flows Case Study
Source: Environ Manage. 2023 May 25;72(4):754–70. doi: 10.1007/s00267-023-01829-6 (PMC10460341; doi:10.1007/s00267-023-01829-6)
Supplement: Supplementary file 1 — Supplementary materials [file 267_2023_1829_MOESM1_ESM.docx]

# Supplementary Materials

## Appendix A: Kaiela (Lower Goulburn) E-Flows Case Study Summary

### Overall Methodology

This e-flows assessment was a departure from the traditional FLOWS methodology used in Victoria, Australia as stakeholder identification and engagement is a key element of the entire process, including model development. Technical elements of the project are still present and are integrated with the participatory workshops, including hydrologic modeling, quantitative model building, and expert-opinion elicitation. The full methodology is presented in Figure 1. A breakdown of the participatory workshops is presented in Table 1. Note, Workshops 3 and 4 were adjusted based on limitation due to the COVID-19 pandemic and associated restrictions in Victoria, Australia.


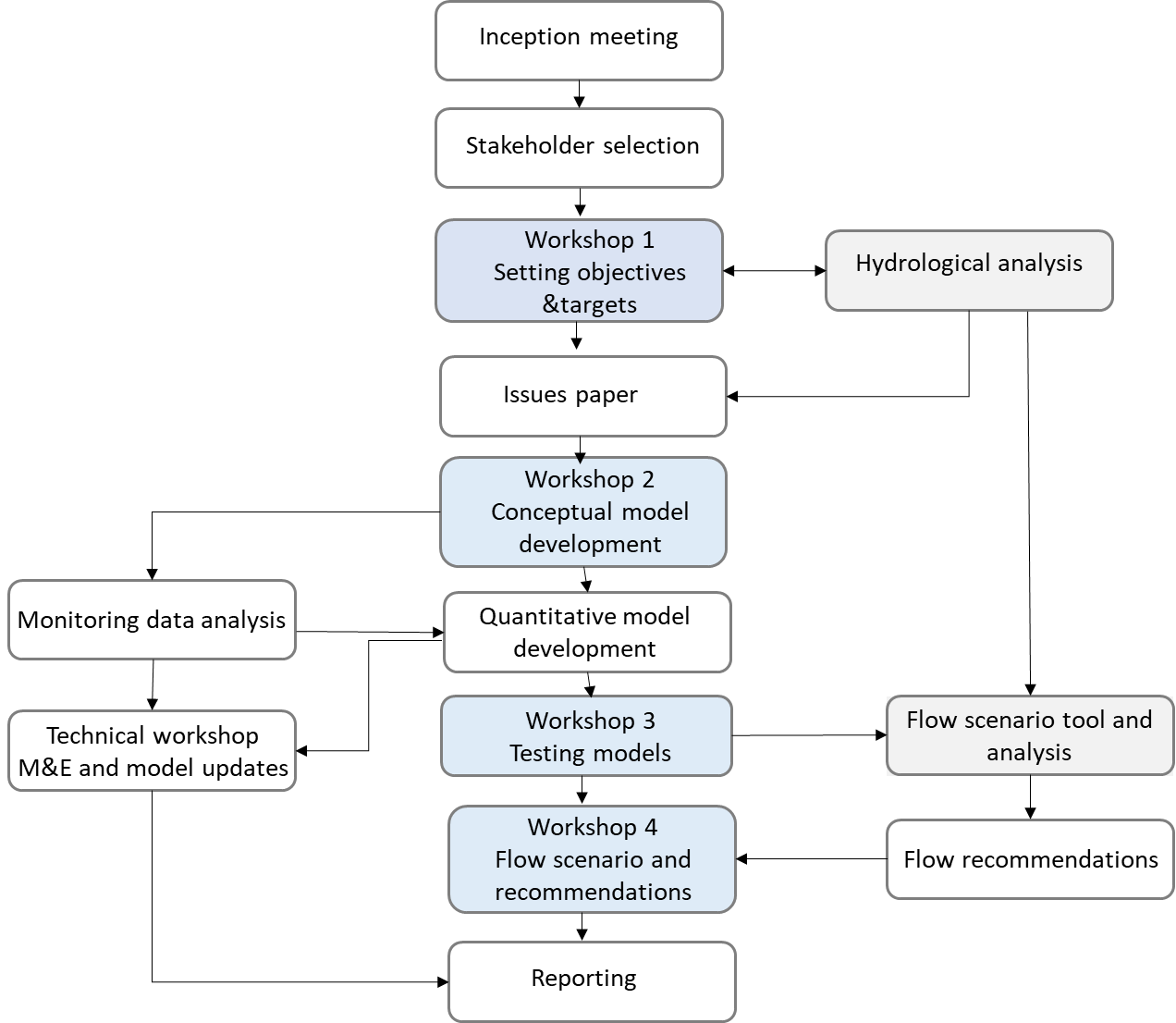


Figure 1: Overview of complete project approach with participatory workshops highlighted. From Horne et al. 2020.

| **Workshop** | **Format and Notes** | **# of Attendees** | **Outcomes** |
| --- | --- | --- | --- |
| *Workshop 1:*  Setting Objectives | Full day in-person workshop with all stakeholder groups.  Breakout groups with activities | 15 (8 participants were apologies and they were contacted following the workshop for updates) | Ecological objectives were selected, as well as *process-objectives* for how decision-making should be handled within the group |
| *Workshop 2:*  Conceptual Model Building | Full day in-person workshop with all stakeholder groups  Breakout groups with activities | 23 | Conceptual models were developed for each of the fundamental objectives identified in the first workshop. These were recorded and used as the basis for quantitative model development |
| *Workshop 3:*  Model Validation and Testing | Originally planned as a full day workshop, this became a half-day online workshop. All stakeholder groups were invited, but only expert panel members attended. Models were additionally tested during individual meetings between the project team and expert panel members | 6 (expert panel members) | Different aspects of the flow regime were changed as inputs into the models and outcomes were observed. Experts validated that models were behaving as expected. In some cases, models were examined for anomalous behavior. |
| *Workshop 4*:  E-flows recommendations and prioritization | Originally planned as a full day workshop, this became a half-day online workshop. This was primarily attended by community members and agency representatives. | 12 (community members and agency reps. Those not in attendance were e-mailed and offered the opportunity to comment) | The project team had prepared a breakdown table of the flow recommendations based on model outputs for the workshop. Recommendations were reviewed one-by-one and comments from participants were recorded by the project team. The final recommendations contained amendments and notes based on this workshop |

### Summary of Fundamental Ecological Objectives from First Workshop

#### Fauna

**Birds:**

- Maximize self sustaining, diverse populations of native wetland bird populations
- Associated means objectives
- Achieve wetland connectivity
- Maintain diverse vegetation
- Maintain appropriate hydrology of wetlands

**Platypus:**

- Maximize self sustaining
- Achieve a viable platypus population. (It is noted that this would also support water rats)

**Turtle**

- Maintain viable population of three species of turtle

**Fish (Small-bodied):**

- Increase abundance and diversity of small bodied native fish
- A sustainable, resilient and diverse small bodied native fish population (resilient is defined as being able to recover or respond to shocks without intervention)

**Fish (Large-bodied):**

- Increase abundant, viable, and diverse populations of large bodied fish.
- Support a diverse, sustainable, and resilient population of large bodied fish.

#### Flora

**Wetland/Floodplain Vegetation:**

- Increase structural complexity and diversity of native floodplain vegetation, including wetlands.
- Support wetland vegetation.
- Support diverse native vegetation in wetlands at multiple levels.
- Increase abundance and diversity of floodplain vegetation

**Instream and Bank Vegetation:**

- Maximize structural complexity and diversity native bank vegetation.
- Increase diversity of native inbank and littoral zone vegetation that is resilient (can cope with sustained or frequent “shocks”).
- Increase diversity of bank and riparian vegetation at multiple levels

### Summary of Fundamental Social Objectives from the First Workshop

#### Community/Social

**Recreational:**

- Ensure that social and community needs of the river are met including fishing, boating, walking, swimming, (WQ S&D??)

**Community Health:**

- Promote community health and well being through connection to river.
- Promote cultural and indigenous values connected to the river.

**Decision-process that builds relationships**

- Promote personal participation towards good outcomes

### Process-Objectives Defined from First Workshop:

Process-objectives from the first workshop were critical for developing an evaluation framework for the participatory process itself. The following process-objectives were used in developing the exit interviews that were assessed to determine participatory success. These objectives were defined during the first workshop with key points arising from conversations that took place during the workshop. These have been reported in the Kaiela (Lower Goulburn) E-Flows Assessment (Horne et al., 2020).

| **Objective** | **Broad Definition** | **Key Points** |
| --- | --- | --- |
| Community Ownership | Developing a sense of community ownership entails identifying opportunities for meaningful community engagement and ensuring that community representation is equitable. For the purpose of decision making, the participatory group has articulated that they find it important for community members to be engaged during the entire course of the project and be “brought along on the journey” of decision making on environmental flows more broadly | • Stakeholders need to have a sense of ownership of decisions  • The decision-making process builds relationships  • Process or journey needs to be oriented to decision making process, not focused only on outcomes  • The decision process needs to meaningfully engage stakeholders so that they don’t feel like they’re being spoken at  • The decision process should include a diverse and representative mix of stakeholders  • Stakeholders are included at all stages of the decision-making process  • The decision process incorporates local knowledge |
| Transparency | Transparency requires detailed and methodological approaches to documenting the decision-making process and communicating this information to community members through appropriate avenues. In follow up conversations after the first workshops, some community members identified the benefit of having one-on-one conversations with agency representatives. | • Information needs to be accessible and well communicated to the community  • The decision process needs to be transparent  • Be transparent about environmental flow components and their targets and benefits  • Be transparent about agency considerations and policy constraints  • There needs to be agency collaboration on decisions |
| Knowledge Exchange | Community members voiced an interest in having the opportunity to engage with scientists and learn about river ecology and needs, while agency representatives would like to better understand community priorities. Community members have their own, unique perspectives and understandings of the river, and their knowledge of the system should not be discounted. | • Information needs to be accessible and well communicated to the community  • The decision process should incorporate multidirectional learning  • Decisions should be informed by the best available science and history  • Identify areas where more research and knowledge is needed (for instance, floodplain specialist species)  • The decision process should incorporate local knowledge  • There needs to be agency collaboration on decisions |

## Appendix B: Guiding Questions for Semi-Structured Interviews

### Introductory Interviews

**Introductory Interview Questions**

***What is the problem and what do you know about it?***

What do you think an environmental flow is trying to achieve?

What are some of the issues or problems around environmental flows in the Goulburn?

***Who is involved in the project and how?***

What is your relationship with the river/catchment? How do you use the river/catchment?

Do you think you are broadly representative of any stakeholder groups?

How would you define your role as a stakeholder? What kind of stakeholder are you?

What do you think the role of stakeholders should be in a participatory approach? And what do you think would make a participatory approach effective?

Are there any individuals or organizations that should definitely be included in this process? Knowing that we have to limit our participatory group, but I can feed this information back to the CMA.

Who is the ultimate decision maker for this project?

***Specific questions about participation and establishing baseline for success of participation***

What do you think has triggered this process? Why did you choose this process?

Why do you think a participatory approach is being used? What issues do you think a participatory approach might address?

Have you been involved in participatory approaches before and how were those experiences?

Do you have any concerns about the participatory approach?

***What is success?***

What would a successful outcome for this project look like for you? For the catchment?

### Exit Interviews

**Exit Interview Questions**

**Transparency**

Were you appropriately briefed about the project and method in the beginning of this project? Did you understand your role in the project as an expert panel stakeholder?

Can you describe your role as an expert panel stakeholder?

Did you understand the time commitment expected for this project?

Do you have a good understanding of the process used to determine the environmental flow recommendations?

Were you adequately updated about progress during the course of the project?

Does the final report accurately represent the methodology as you understand it? In particular, do you believe the report accurately describes the model development process and captures the uncertainties and concerns regarding your area of expertise?

Do you feel you have a good understanding of how the environmental flow recommendations will be applied in the future?

Has your understanding of environmental flow decision making improved? For instance, do you feel you understand how the catchment management authorities make their yearly watering decisions?

**Community Ownership**

Was there an appropriate representation of stakeholders during the workshops? Were the majority of interested parties and interests represented?

If a perspective or group was missing from these workshops, can you elaborate? Who should have been included that wasn’t?

Do you feel that this process reflected the values of the community? Were the values of the community stakeholders used as a starting point for the process before technical elements were brought in?

Did you feel the level of involvement for community members was appropriate for each stage of this process?

Did you feel the level of involvement for researchers was appropriate for each stage of this process?

Did you feel the level of involvement for water managers was appropriate for each stage of this process?

Did these workshops help foster community relationships between stakeholders? Can you elaborate? If not, did these workshops weaken community relationships and why?

**Knowledge Exchange**

Which of the workshops were you able to attend?

*Nov 19, 2019- Workshop 1: Objective Setting (Full day workshop in person)*

*Dec 11, 2019- Workshop 2: Conceptual Models (Full day workshop in person)*

*March 24, 2020- Technical Workshop with expert panel demonstrating first drafts of models (Half day workshop on zoom)*

*Jan-April 2020- Bayesian Model Development (Expert Opinion Elicitation and one on one meetings with model experts)*

*23 June 2020- Environmental Flows Workshop for technical panel demonstrating models and flow recommendations (Half Day, zoom)*

*June-August 2020- Review of final report and recommendations by all stakeholders*

Did you feel comfortable expressing your perspective and knowledge during the workshops? If not, why not?

Do you feel that your input was valued during workshops (both community and expert panel workshops)?

Was there adequate opportunity to ask questions and discuss with other stakeholders and experts about the topic at hand?

Did you feel the best available science was used to inform model development and the flow recommendations?

If not, can you describe what you would change about the process regarding model development?

Did you understand how the larger stakeholder workshops fed into model development? Did the stakeholder workshops have a significant influence on the ultimate model structure?

Did the larger stakeholder workshops and the expert elicitation complement each other?

Were the scientific and technical elements of this project adequately communicated to the non-expert stakeholders?

Were the appropriate agency representatives present? If not, who was missing?

How would you recommend we make the results of this project accessible to the wider community?

How would you describe the communication between the University of Melbourne team and the expert panel?

**Overall Success**

Would you say that you have overall had a positive or negative experience through your engagement with this project? How has this experience compared to previous involvement in environmental flows projects?

Any other comments or recommendations if we were to do this again?

What surprised you most about this project?

How would you summarize COVID’s impacts on this project?

Did the project team adequately respond to these impacts and communicate the adjusted plan?

Do you have recommendations for how we could have improved our response to the impacts of COVID?
